# Nicotine is associated with smoking dependence and vascular inflammation through cotinine: A mediation analysis

**DOI:** 10.18332/tid/171356

**Published:** 2024-01-19

**Authors:** Kumboyono Kumboyono, Indah N. Chomsy, Nadya N. Shalshabilla, Hidayat Sujuti, Arie Srihardyastutie, Cholid T. Tjahjono, Teuku Heriansyah, Titin A. Wihastuti

**Affiliations:** 1 Department of Nursing, Faculty of Health Sciences, University of Brawijaya, Malang, Indonesia; 2 Doctoral Program of Medical Science, Faculty of Medicine, University of Brawijaya, Malang, Indonesia; 3 Master Program of Biomedical Science, Faculty of Medicine, University of Brawijaya, Malang, Indonesia; 4 Department of Biomolecular-Ophtalmology, Faculty of Medicine, University of Brawijaya-Saiful Anwar General Hospital, Malang, Indonesia; 5 Department of Chemistry, Faculty of Mathematics and Natural Science, University of Brawijaya, Malang, Indonesia; 6 Department of Cardiology and Vascular Medicine, Faculty of Medicine, University of Brawijaya-Saiful Anwar General Hospital, Malang, Indonesia; 7 Department of Cardiology and Vascular Medicine, Syiah Kuala University, Banda Aceh, Indonesia

**Keywords:** nicotine, cotinine, vascular inflammation, oxidative stress, tobacco-induced disease

## Abstract

**INTRODUCTION:**

The main alkaloid component in cigarettes is nicotine. Cotinine, a metabolite of nicotine, is capable of causing dependence effects through endless mechanisms modulated by the ion channel nicotinic acetylcholine receptors nAChRs. Nicotine and cotinine can also cause damage to blood vessels through a chronic inflammatory process mediated by the Ligand-Tie2 Angiopoietin Receptor system. Hypoxic conditions that occur due to vascular inflammation cause a decrease in the concentration of nitric oxide (NO). This study aimed to evaluate the relationship between NO levels and cotinine through the expression of nAChRs that mediate the nicotine dependence mechanism and Tie2 (Tyrosine Kinase 2) expression.

**METHODS:**

A cross-sectional study was conducted with 200 participants grouped into two groups based on their smoking status: 100 smokers and 100 non-smokers. All participants were men aged 20–40 years with no history of cardiovascular disease, diabetes mellitus, or dyslipidemia, and were not currently on medication. According to the parameters used, all blood samples were taken from peripheral blood for analysis using the ELISA kit or Colorimetric Assay Kit.

**RESULTS:**

Cigarette consumption increases blood cotinine concentrations in smokers and causes dependence by modulating nAChRs. The study indicates an emerging cycle regarding nicotine–cotinine consumption and nAChRs expression. In addition, the data in this study showed a significant relationship (p<0.001) regarding the cycle formed with decreased NO levels as a result of damage caused by Tie2-mediated inflammation.

**CONCLUSIONS:**

There is a relationship between NO levels and cotinine through nAChRs, which mediate the nicotine dependence mechanism and Tie2 expression.

## INTRODUCTION

Cotinine is the primary alkaloid component found after the metabolism of nicotine^1-3^. Nicotine and cotinine are two substrates strongly suspected to be responsible for dependence on smoking. This dependence is mediated by ion channel nicotinic acetylcholine receptors (nAChRs)^1,4,5^. The role of cotinine, which is known to be a weak agonist in the α3/α6β2, α4β2, and α7 nAChRs still raises many different opinions among researchers. Tan et al.^1^ stated that cotinine has a lower ability to penetrate the blood–brain barrier, so it takes ten times the nicotine concentration to cause the same effect on nAChRs. However, research has reported that cotinine is a whole agonist component of the seven mutant nAChRs in the human brain^1^. Therefore, it is necessary to investigate the role of cotinine as an agonist of nAChRs in the mechanism of nicotine dependence.

Nicotine and cotinine levels that continue to increase in the body of smokers will harm many organs through the inflammatory response^6^. One of these inflammatory responses is mediated by the Angiopoietin Ligand-Tie2 Receptor (Tie2) system^7^. Angiopoietin 2 (Ang2) is an angiopoietin family with adverse effects such as plasma leakage, vascular growth regression, and endothelial cell death^7,8^. Furthermore, the inflammatory response will cause tissue hypoxia and increase levels of Ang2^7,9,10^. Increased levels of Ang2 can sensitize endothelial cells to TNF-α, produce ROS, increase endothelial cell permeability, reduce the number of endothelial cells, and interfere with blood vessel formation^6-10^. Studies indicate that hypoxic conditions could induce increased expression of Tie2 receptors in the bovine aorta, human umbilical vein, and endothelial cells in small blood vessels.

Another effect of oxidative stress and inflammation caused by nicotine and cotinine is the increased activity of endothelial nitric oxide synthase (eNOS) to synthesize nitric oxide (NO), which plays a vital role in inhibiting the formation of ROS^11^. A further impact of oxidative stress on endothelial cells is increased activity of eNOS and NO levels^11^. Furthermore, Kumboyono et al.^6^ stated that damage to endothelial cells due to chronic smoking would decrease NO levels because of endothelial cell degradation. This study aimed to evaluate the relationship between NO levels and cotinine through the expression of nAChRs that mediate the nicotine dependence mechanism and Tie2 expression. This mechanism is needed for the early detection of endothelial cell disorders before fatal blood vessel damage due to smoking.

## METHODS

### Study design and participants’ characteristics

This was a cross-sectional study. The sample size was calculated using G*Power 3.1 software for the difference between two independent means (two groups) using effect-size (d) 0.5; error probability (α) 5%; power (1-β error probability) 95%; and allocation ratio n2/n1=1. Based on these calculations, a minimum sample size of 88 was obtained for each group. Thus, the minimum sample number was 176 participants.

A purposive sampling method was conducted on 315 participants from the Faculty of Medicine, University Brawijaya and Saiful Anwar General Hospital Malang. A total of 200 participants were divided into two groups using purposive sampling, 100 people each, namely smokers and non-smokers. The sampling method is shown in Supplementary file Figure 1. The participants were given a google form comprising the objectives, benefits, rights, and obligations of participants, as well as research procedures. The informed consent was provided electronically only if the participant had comprehended and acknowledged the information.

A smoker was defined as someone with a smoking history of more than a year with mild cigarettes, kretek, or electric cigarettes. A non-smoker was defined as someone who had never smoked or quit smoking for more than a year. The participants of both groups should have no history of cardiovascular and metabolic diseases such as hypertension, dyslipidemia, obesity, diabetes mellitus, infection, and cancer. The medical records of participants were collected to obtain the history of diseases. This study used only men as participants due to the involvement of sex hormones in nicotine metabolism^12^. CYP2A6, the primary enzyme for nicotine metabolism, is induced by estrogen via ERα^12^. Thus, women metabolize nicotine faster than men^12,13^. This finding has been supported by previous studies that showed the ratio of cotinine/nicotine in plasma was higher in women than in men^14^. The clearance of nicotine and cotinine has also been reported to be significantly higher in women than in men (13% and 24%, respectively)^13^; oral contraceptive use further accelerates nicotine and cotinine clearances by 28% and 30% respectively, compared to women who are not using oral contraceptives^13^. The inclusion criteria of the participants were: male, aged 20–40 years, and not taking drugs at the time of the study.

### Data collection procedure

Participants who fulfilled the inclusion criteria were examined for their vital signs (blood pressure, pulse, respiration, and body temperature). The laboratory analysts were blinded to each participant’s smoking status while collecting and analyzing the blood samples.

### Enzyme-linked immunosorbent assay to analyze the level of cotinine, nAChRs, and Tie2

Blood samples were taken at the Central Laboratory of Saiful Anwar Hospital; 2.5 mL of peripheral blood was taken from each participant according to the venipuncture procedure. Then, the blood was put into a tube containing ethylene diamine tetra acetic (EDTA). Cotinine level, nAChRs, and Tie2 measurements were conducted by centrifuging the blood for 15 min at a speed of 2000–3000 rpm at a temperature of 2–8^o^C to collect the plasma. Then, the samples were analyzed using the ELISA Kit to determine the levels of cotinine (Human Cotinine, Bioassay Technology Laboratory, E2043Hu), nAChRs (Human Nicotinic Acetylcholine Transferase Receptor, Bioassay Technology Laboratory, E1330Hu), Tie2 (Human Tyrosine Kinase with Immunoglobulin-like and EGF-like Domains 2, Bioassay Technology Laboratory, E1219Hu)^15^.

### Colorimetric assay to analyze the level of nitric oxide

NO level was determined using 2.5 mL of blood samples put into an EDTA tube and centrifuged for 10 min at a speed of 2000–3000 rpm at a temperature of 4°C to collect the plasma. The plasma was analyzed using the colorimetric kit to assess the NO level (Nitric Oxide Colorimetric Assay Kit, Elabscience, E-BC-K035-S). Another study by Xiong et al.^16^ using the same analysis method to determine NO concentration showed the result is significant (p<0.01) in their analysis.

### Data analysis

Normal distribution and equality of variances analysis was carried out as a requirement for subsequent data analysis. The normal distribution of the sample was tested with the parametric test Kolmogorov-Smirnov (α>0.05). The equality of variances for each variable was tested with Levene’s test. Following this, we used Partial Least Square Structural Equation Models (PLS-SEM), a regression technique to address each parameter’s direct and mediating effect for smoke dependence and vascular inflammation in linear relationships. The independent variable’s mean and standard deviation values were analyzed using WarpPLS version 7.0. Path diagrams were used to portray corresponding PLS-SEM, with square boxes representing measured parameters, single-headed arrows representing direct relationships, and single-headed dashed line arrows representing indirect relationships. Cotinine and Tie2 were used as the primary mediator parameters of nicotine and nAChRs’ biological effects on smoke dependence and vascular inflammation. Validation of the structural model was validated using goodness of fit to carry out the path coefficient, coefficient of determination (R^2^), and cross-validated redundancy measure (Q^2^).

All the data were distributed normally and homogenously. The path coefficient was the essential criterion used to calculate the relationship between variables. The value used as a reference was described previously. R-squared (R^2^) as the predictive power of endogenous variables was everywhere between 0 and 1. An R^2^ value of 0.67 is considered strong, 0.33 moderate, and 0.19 weak. The Q^2^ is used to see the predictive relevance of the research model. A Q^2^ value greater than zero means that the model has predictive relevance.

Hypothesis analysis of the direct effect of the variable was carried out to test the path coefficient and whether there is a direct effect of the exogen variable on the endogen variable. The test criteria are based on p-value ≤ level of significance (α=5%), then it is stated that the exogen variable has a significant effect on the endogen variable. The indirect effect hypothesis analysis was conducted to test whether the exogen variable’s indirect effect on the endogen variable is through the mediator variable. The test criteria are based on p-value ≤ level of significance (α=5%), then it is stated that the exogen variable has a significant effect on the endogen variable through the mediator variable.

## RESULTS

The characteristics of the participants in this study are shown in Table 1, with the highest age distribution at 25–30 years. Additional questions were asked in the smoker group to obtain information about the type of cigarette, length of time smoking, and the level of nicotine dependence, as previously measured using the Fagerström questionnaire. Table 1 also shows that 50% of the participants used mild cigarettes, and 36% have smoked for 6–10 years. Presumably, smokers aged 20–40 years have started smoking in childhood and adolescence. The result of the Fagerström questionnaire showed that 31% of smokers were highly dependent on nicotine.

**Table 1:** Baseline characteristics of the participants (N=200)

*Characteristics*	*n*	*%*
**Smoking status**		
Non-smoker	100	50
Smoker	100	50
**Age** (years)		
20–25	56	56
26–30	58	58
31–35	46	46
36–40	40	40
**Type of cigarette**		
Mild	50	50
Kretek	30	30
Electronic	20	20
**Nicotine dependence**		
None	15	15
Minimal	20	20
Minimal to moderate	15	15
Moderate	19	19
High	31	31
**Smoking duration** (years)		
≤5	28	28
6–10	36	36
11–15	20	20
16–20	15	15
>20	1	1

**Figure 1:**
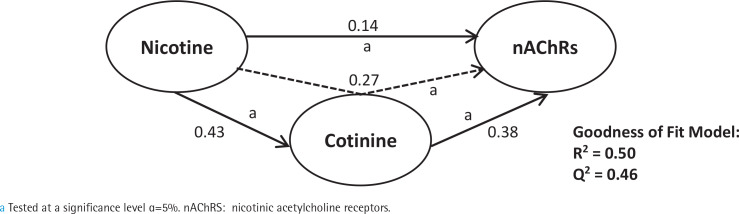
Pathway modelling of inter-relationships between nicotine, cotinine, and nAChRs. The direct and indirect coefficient values allow comparison between the strength in each model. Nicotine is a moderate exogenous predictor for nAChRs variation both directly and indirectly through cotinine. Single-headed arrows indicate direct relationships, and single-headed dashed line arrows indicate indirect relationships

**Figure 2:**
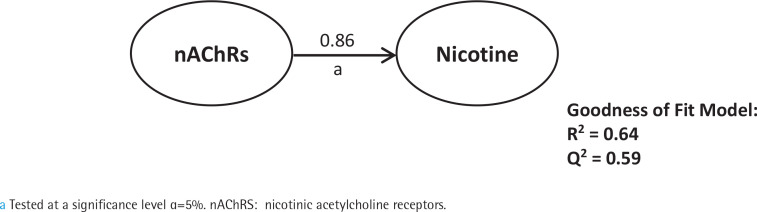
Pathway modelling of inter-relationships between nAChRs and nicotine. The direct effects of nAChRs in contributing to nicotine levels

**Figure 3:**
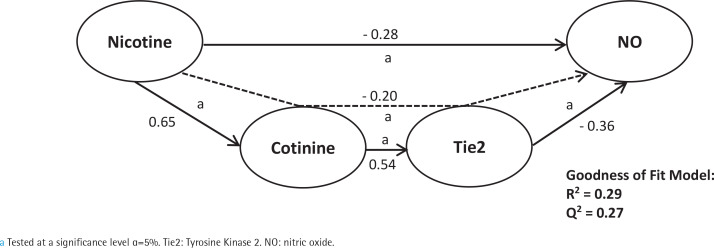
Pathway modelling of inter-relationships between nicotine, cotinine, Tie2 and NO. The direct and indirect coefficient values allow comparison between the strength in each model. Mediation models for Tie2 and NO as endogenous vascular inflammation parameters as described in detail in the manuscript. Single-headed arrows indicate direct relationships, and single-headed dashed line arrows indicate indirect relationships

**Figure 4:**
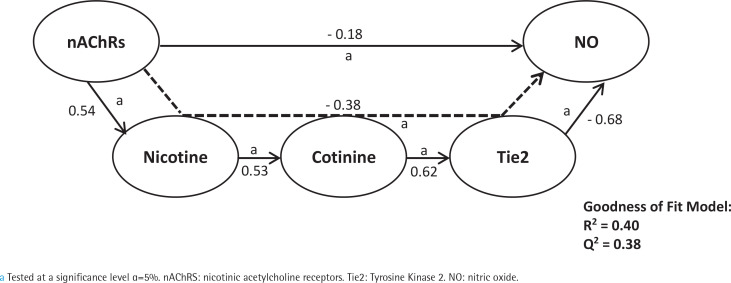
Pathway modelling of inter-relationships between nAChRs, nicotine, cotinine, Tie2, and NO. The direct and indirect coefficient values allow comparison between the strength in each model. Mediation models for nAChRs as described in detail in the manuscript. Single-headed arrows indicate direct relationships, and single-headed dashed line arrows indicate indirect relationships

First, we analyzed smoking dependence mechanisms’ exogen and mediating variables (Figures 1–4). Figure 1 shows that nicotine as an exogenous and cotinine as mediating variable has a moderate predictive power of smoking dependence upon nAChRs (R^2^=0.50; Q^2^=0.46; p<0.05). This model also showed that cotinine is directly associated with nAChRs (path coefficient=0.38; p<0.05). Figure 2 measures the direct effect of nAChRs upon nicotine independently with a moderate prediction (path coefficient=0.86; R^2^=0.64; Q^2^=0.59; p<0.05). The same analysis was done to measure the exogen and mediating variables of vascular inflammation depicted in Figures 3 and 4. Figure 3 represents nicotine as an exogen variable that significantly lowered NO levels both directly and indirectly through cotinine and Tie2 as mediating variable with a weak prediction (direct coefficient= -0.28; indirect coefficient= -0.20; R^2^=0.29; Q^2^=0.27; p<0.05). The difference between Figure 3 and Figure 4 is the involvement of nAChRs in contributing to the cyclic process of nicotine dependence and vascular inflammation. Figure 4 portrays nAChRs as an exogen variable that was able to predict moderately upon low NO level significantly (direct coefficient= -0.18; indirect coefficient= -0.38; R^2^=0.40; Q^2^=0.38; p<0.05).

**Figure 5:**
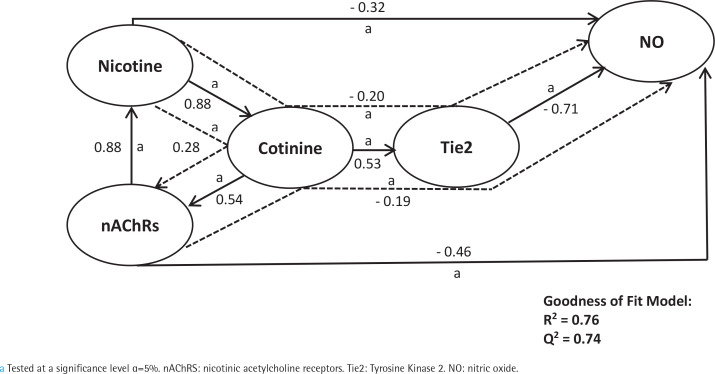
The final structural model summarizes nicotine’s contribution to smoking dependency and vascular inflammation through cotinine. The direct and indirect coefficient values allow comparison between the strength in each model. This model shows a strong prediction that nicotine contributes directly and indirectly to predicting smoking dependency and vascular inflammation. Single-headed arrows indicate direct relationships, and single-headed dashed line arrows indicate indirect relationships

Second, Figure 5 summarizes the mediation model of nicotine upon smoke dependence and vascular inflammation via the same parameters (Figure 5). This model depicted that nicotine strongly predicts smoke dependence indirectly via cotinine (indirect coefficient=0.28; R^2^=0.76; Q^2^=0.24; p<0.05). The same results could be seen in nicotine contribution to strongly predict low NO levels directly and indirectly via cotinine and Tie2 (direct coefficient= -0.32; indirect coefficient= -0.20; R^2^=0.76; Q^2^=0.74; p<0.05).

The results of the direct and indirect effects of the independent variable are depicted in Figures 1–5. Each exogen variable significantly impacts endogen variables based on the value of α<5%. There is a significant positive correlation between variables except for Tie2 and NO, which showed a negative correlation. It can be said that the upregulation of Tie2 expression significantly reduced the level of NO.

## DISCUSSION

A study on nicotine and cotinine is essential because of their far-ranging effects on various diseases. This study discussed the relationship of cotinine levels with nicotine dependence and the decrease in NO levels through the expression of nAChRs and Tie2 receptors. Explaining this phenomenon is essential for the primary prevention of vascular damage due to smoking behavior.

### Cotinine as nAChRs agonist

Previous studies have discussed the role of nicotine in contributing to smoking dependence. Meanwhile, the role of cotinine as the major metabolite of nicotine with a 19–24 h plasma half-life is still controversial about its involvement in mediating smoking dependence^1,3^. This study clarified that cotinine had a significant correlation (p<0.001) in increasing the nAChRs expression as a marker of nicotine dependence. The upregulation of nAChRs indicates that cotinine can desensitize nAChRs. Thus, more functional receptors are needed to produce its addictive effects for smokers. Dwoskin supports this phenomenon: an increase in dopamine firing and flow in rat striatal after cotinine administration. However, it is only temporary, and the dopamine flow will gradually decrease after continuous cotinine administration^1^. Another study showed that cotinine administration increased α4β2 nAChRs expressions in undifferentiated mice 2a neuroblastoma cells^1^.

nAChRs are the primary receptor in accommodating the mechanism of nicotine dependence in smokers^1,4,5^. α4β2 and α7 subtype receptors are the most abundant receptors in the human brain and modulate cognitive, affective, and pain processes^17^. In this study, a significant increase in nAChRs (p<0.001) showed its role in mediating nicotine dependence. Various subtypes of nAChRs are expressed in the mesolimbic pathway, especially in the ventral tegmental area (VTA) and nucleus accumbens (NAs)^5^. The binding of nicotine or cotinine with nAChRs may activate the dopamine neuron, causing cation influx, and thus, the neuron is depolarized^5^. As a result, there is a spike in dopamine firing in NAs^5^. Chronic exposure to nicotine and cotinine will desensitize nAChRs, causing withdrawal symptoms due to a lack of nicotine in the blood. This activation of nAChRs will cause individuals to fall into dependence.

### Cotinine and Tie2 receptor expression

Accumulating nicotine, cotinine, and other cigarette components in the smoker can affect endothelial cell function^6^. The presence of inflammation on endothelial cells will activate the endothelial cells, which causes the accumulation of inflammatory cells at the site, and the environment becomes hypoxic^7,9^. Ang2/Tie2 is a known pathway to modulate endothelial cell remodeling in inflammatory and hypoxic conditions^7,9,13^. This study showed that cotinine level was able to increase the expression of Tie2 receptors significantly (p<0.001). Similar results were shown by Willam et al.^18^ who reported that hypoxic conditions induced and increased Tie2 receptor expression in the bovine aorta, human umbilical veins, and endothelial cells in small blood vessels.

### Tie2 receptors expression and changes in NO levels

The Tie2-Ang2 complex will cause plasma leakage, regression of vascular growth, and endothelial cell death^7,8^. Persistent inflammation on endothelial cells may promote eNOS activity to produce NO, known for its role as an anti-inflammatory and antioxidant agent^11,14^. It was presumed that inflammation mediated by the Ang-Tie system would increase NO levels as a form of homeostasis to prevent platelet aggregation and continuous leukocyte adhesion on the arterial walls^11^. However, in this study, a significant increase in Tie2 expression was linked to lower NO levels in the blood (p<0.001), which can be concluded that Tie2 expression modulates the inflammation process. The mechanism of decreasing NO levels in severe endothelial cell damage due to chronic inflammation has yet to be ascertained^14^. Some of the mechanisms that support this are: 1) membrane receptor damage on endothelial cells that could interfere with the interaction with NO stimuli, 2) the loss of eNOS substrates, 3) low NOS concentration or activity, 4) disruption of NO production in the endothelium; and 5) elevated NO degradation due to free radicals exposure^19^. In complement to oxidative stress, the endothelial cells’ ability to recognize their environment and their activity in producing NO has been lost^20^. Thus, this occurrence will further damage endothelial cells in the future.

### Limitations

This study cannot be generalized to women and passive smokers. Moreover, this study only focused on detecting the endothelial cell membrane receptor that modulates inflammation, Tie2. However, in other studies, Tie2 has always been discussed and analyzed with its ligand, Angiopoietin. So further research is needed to measure the mediating role of Tie2 and Angiopoietin in vascular inflammation in smokers. This research has limitations in revealing causal relationships, either directly or indirectly. Thus, it is necessary to carry out further research with a causal design.

### Implications

This research can provide new certainty to health workers and the public regarding the short- and long-term effects of consuming tobacco. Trends in society today have shown a shift in the age of smokers, where it can be found that smoking begins in children aged 12 years and adolescents. The short-term effect is that individuals become dependent due to the nicotine and cotinine content. This study has proven that the consumption of nicotine and cotinine will lead to a cyclical chain in which smokers will tend to continue their smoking behavior. This tendency is caused by the ‘beneficial’ and pleasurable effects of the accumulation of nicotine and cotinine, so smokers will always try to get these effects by continuing to smoke. The individual may increase the frequency and intensity to get more rewardable effects. In addition, the long-term risks and side effects that can be found due to the accumulation of nicotine and cotinine, are the occurrences of blood vessel damage through the mechanism described above.

This research can be used by the government, health workers, and the community to start implementing regulations regarding tobacco consumption. Individuals who try to start smoking or who already smoke will inevitably lead to a state of dependence through the mechanisms described previously. It is essential to pursue a tobacco consumption prevention program to eliminate the short- and long-term effects of smoking. In addition, in individuals who have smoked, risk and side effects analysis can be carried out through the measurement of variables in this study so that it can be a reference in considering smoking cessation programs.

## CONCLUSIONS

Smoking behavior forms an endless loop between nicotine, cotinine, and nicotine dependence, which causes individuals to continue to smoke. The dependence makes it difficult for a person to quit smoking and increases the risk of vascular inflammation from the early stages of smoking. Therefore, an integrated policy is needed for smoking prevention, early detection of diseases caused by smoking and smoking cessation to avoid the worsening of inflammation of blood vessels which is fatal to the cardiovascular system, in the following years.

## Data Availability

The data supporting this research are available from the authors on reasonable request.
