# Supplementary figures and images for: Nicotine is associated with smoking dependence and vascular inflammation through cotinine: A mediation analysis
Source: Tob Induc Dis. 2024 Jan 19;22:10.18332/tid/171356. doi: 10.18332/tid/171356 (PMC10798223; doi:10.18332/tid/171356)

Supplementary file Figure 1

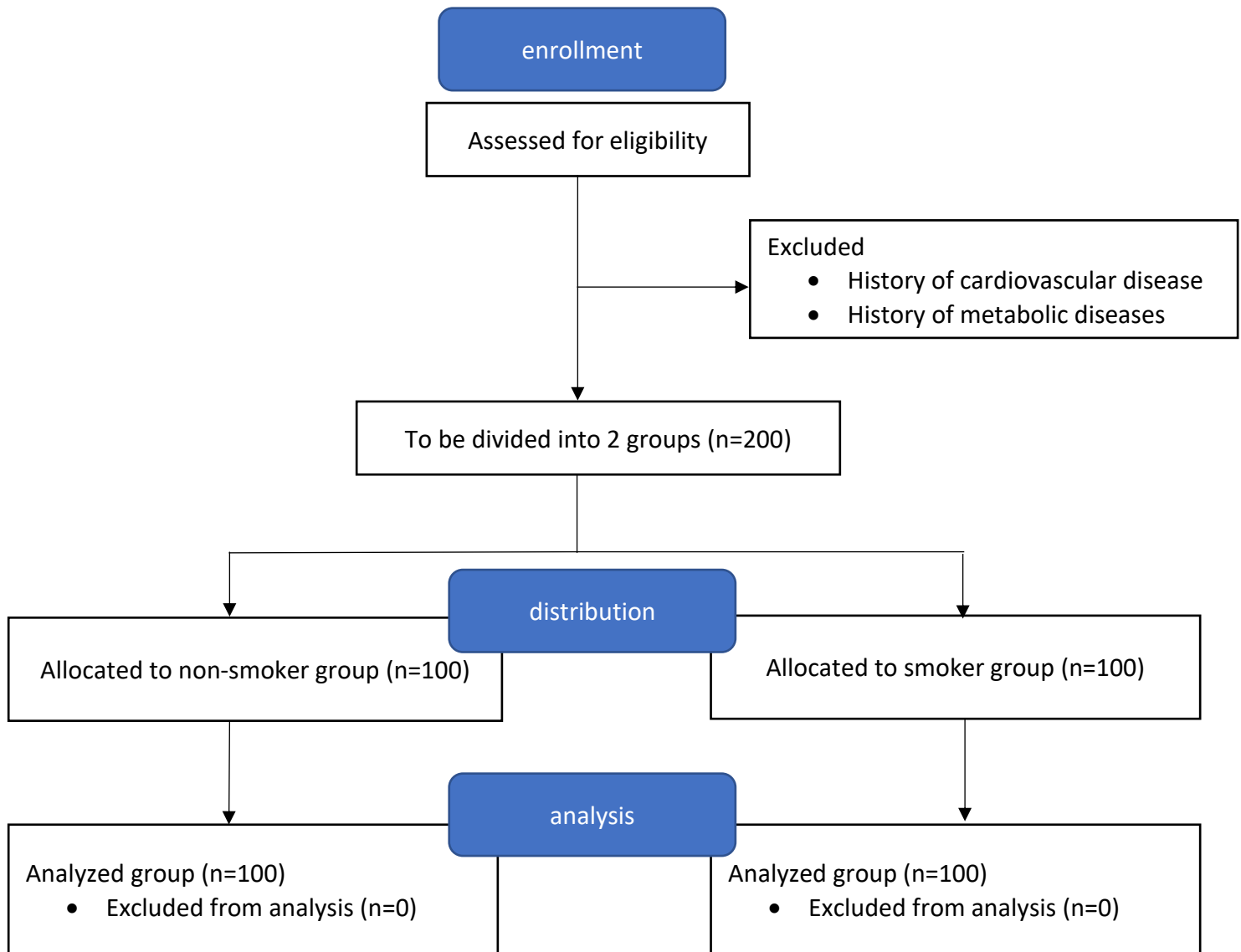

Supplement: Supplementary file 1 [file TID-22-16-s1.pdf]
